# KCNE2 and gastric cancer: bench to bedside

**DOI:** 10.18632/oncotarget.7921

**Published:** 2016-03-04

**Authors:** Geoffrey W. Abbott, Torsten K. Roepke

**Affiliations:** Bioelectricity Laboratory, Department of Pharmacology and Department of Physiology and Biophysics, School of Medicine, University of California, Irvine, CA, USA

**Keywords:** potassium channel, KCNQ1, KCNE2, gastric cancer, 5-Fluorouracil

Gastric cancer (GC) is one of the most common cancers worldwide. Among GC patients the mortality rate remains high - most are diagnosed at advanced disease stages due to the lack of symptoms in early disease. Prominent risk factors include age, male sex, smoking, radiation, *H. pylori* colonization, and a positive family history. Depending on tumor staging, treatment consists of neoadjuvant, adjuvant, and palliative therapy combining surgery and radiochemotherapy. 5-Fluorouracil, the first-line chemotherapeutic agent for metastatic GC, is ineffective in many patients, and methods to reliably distinguish patients that are resistant to 5-Fluorouracil-based regimens, *versus* those that might benefit, are urgently warranted 1. In the Vol.7, No.8 of *Oncotarget,* Li and colleagues describe a novel approach employing gene expression profiles to identify a prognostic molecular signature in GC patients. The authors identify and validate two gene-pairs (*KCNE2* and *API5*, *KCNE2* and *PRPF3*) that correlate with GC prognosis after 5-Fluorouracil chemotherapy 2. The significance of this discovery comes more sharply into focus when one considers the already rich literature pertaining to the physiology and pathophysiology of *KCNE2* in the gastric epithelium.

KCNE2 is a relatively promiscuous, single transmembrane-spanning, voltage-gated potassium channel ancillary (β) subunit that is expressed in human heart but is also particularly enriched in certain secretory epithelia, including gastric parietal cells. In parietal cells, KCNE2 forms complexes with the KCNQ1 voltage-gated potassium channel pore-forming (α) subunit, resulting in a remarkable transformation. Homomeric KCNQ1 channels are voltage-gated, i.e., activated by membrane depolarization, and are inhibited by low extracellular pH - not very helpful in the non-excitable parietal cell, which is exposed at its apical side to the low pH of the stomach lumen. However, KCNE2 converts KCNQ1 to a potassium channel that is constitutively open over the range of membrane potentials exhibited by parietal cells. Furthermore, KCNQ1-KCNE2 channel activity is potentiated by extracellular protons. KCNQ1-KCNE2 channels are thus able to function in non-excitable parietal cells, facing the acidic stomach lumen, and there they provide a K^+^ recycling pathway essential for function of the apical gastric H^+^/K^+^-ATPase that acidifies the stomach 3.

That KCNE2 is essential for parietal cell function is evidenced very well by findings from the *Kcne2*−/− mouse line. *Kcne2* deletion essentially eliminates gastric acid secretion in mice, leaving their isolated parietal cells unresponsive to secretagogues and unable to secrete protons after proton loading. *Kcne2*−/− *ex vivo* mouse stomachs remain above pH 6.5 even when stimulated 4. What happens as *Kcne2*−/− mice begin to age is just as striking, and highly pertinent to the recent findings of Li and colleagues 2. By 12 months of age, *Kcne2*−/− mouse stomachs reach six-fold their normal size because of hyperplasia, and the mice universally develop the preneoplastic condition, gastritis cystica profunda, in which large cysts develop associated with herniated glandular profiles in the gastric submucosa. Increased Ki67 and nuclear Cyclin D1 expression, together with TFF2- and cytokeratin 7-expressing metaplasia, are also present in the gastric mucosa of all *Kcne2*−/− mice of at least 1 year of age; some *Kcne2*−/− mice also exhibit pyloric polypoid adenomas and neoplastic invasion of blood vessels in the submucosa 5. In addition to the findings from mice, KCNE2 protein expression was also shown to be downregulated in human gastric carcinoma and adenocarcinoma 5, and in both adenocarcinoma and at the edges of cysts in a patient who developed gastric adenocarcinoma subsequent to gastritis cystica profunda 6.

We do not yet fully understand the mechanisms underlying association of low KCNE2 expression with progression of GC. Chronic achlorhydria, as is caused by *Kcne2* deletion in mice, can predispose to GC, therefore one might predict that this is a major part of the mechanism, at least in mice. However, in addition, KCNE2 may play other roles in cell cycling or proliferation. Tellingly, an earlier report identified KCNE2 as being downregulated in GC tissues and cell lines, and showed that KCNE2 overexpression effectively suppresses SGC7901 cell growth in soft agar and its tumorigenicity in nude mice, contexts in which gastric acidification is irrelevant 7. One possibility is that loss of KCNE2 expression in GC contributes to aggressive growth of gastric tumors because of particularly extreme cell cycle dysregulation, enabling cells in these tumors to proliferate to the extent that they outpace the effects of 5-Fluorouracil, while not being technically resistant to 5-Fluorouracil. The idea of low KCNE2 expression being associated with hyperproliferation is also consistent with the fact that low KCNE2 expression is observed specifically at the edges of cysts in human gastritis cystica profunda 6. Future work will elucidate whether KCNE2 is a druggable target in GC and other cancers, in addition to its promise as a stratification marker for 5-Fluorouracil responsiveness. In these efforts, it will be important to consider discoveries such as the value of KCNE2 as a prognostic marker, in the context of earlier work that already identified the link between KCNE2 and GC, and potential underlying mechanisms.
